# Comparing Groups of Independent Solvers and Transmission Chains as Methods for Collective Problem-Solving

**DOI:** 10.1038/s41598-020-59946-9

**Published:** 2020-02-20

**Authors:** Kyanoush Seyed Yahosseini, Mehdi Moussaïd

**Affiliations:** Center for Adaptive Rationality, Max Planck Institute for Human Development, Berlin, Germany

**Keywords:** Cultural evolution, Psychology, Human behaviour

## Abstract

Groups can be very successful problem-solvers. This collective achievement crucially depends on how the group is structured, that is, how information flows between members and how individual contributions are merged. Numerous methods have been proposed, which can be divided into two major categories: those that involve an exchange of information between the group members, and those that do not. Here we compare two instances of such methods for solving multi-dimensional problems: (1) transmission chains, where individuals tackle the problem one after the other, each one building on the solution of the predecessor and (2) groups of independent solvers, where individuals tackle the problem independently, and the best solution found in the group is selected afterwards. By means of numerical simulations and experimental observations, we show that the best performing method is determined by the interplay between two key factors: the individual’s degrees of freedom as an aspect of skill and the complexity of the problem. We find that transmission chains are superior either when the problem is rather smooth, or when the group is composed of rather unskilled individuals with a low degree of freedom. On the contrary, groups of independent solvers are preferable for rugged problems or for groups of rather skillful individuals with a high degree of freedom. Finally, we deepen the comparison by studying the impact of the group size and diversity. Our research stresses that efficient collective problem-solving requires a good matching between the nature of the problem and the structure of the group.

## Introduction

Collective problem-solving and the related concepts of swarm intelligence and collective intelligence have been studied in a wide variety of domains. In biological systems, examples include the nest construction in eusocial insects^1,2^ or collective foraging in group-living species^3^. In robotics and artificial intelligence, swarms of relatively simple agents can explore and solve optimization problems efficiently^4,5^. Likewise, humans can solve problems in groups during discussions^6^, by means of wisdom of crowds procedures^7^, or when creating Wikipedia articles^8,9^. Despite this considerable diversity of examples and application domains, many instances of collective problem-solving come down to one central challenge: When given a specific number of individuals with a certain skill set, how should a group be structured to produce the best possible collective output?

Numerous procedures have been proposed to that end. These can be divided into two major categories^10,11^: (1) those that involve an exchange of information between the group members, and (2) those that do not.

In the first category, direct or indirect interactions among individuals can lead to the emergence of a collective solution^12–14^. With direct interactions, group members exchange information directly via physical signals. Group-living animals, for example, communicate by means of acoustic and visual cues to detect and avoid predators^15–17^. In human groups, the most common case of direct interaction for solving problems takes the form of group discussions^18,19^, where all group members can freely share ideas and strategies to tackle a problem. This approach can produce good results^20–23^, but is also subject to numerous detrimental effects such as opinion herding, groupthink, and the hidden profile effect^18,19,24^. Also, direct interaction in humans becomes difficult to apply when groups are too large, when members do not work at the same time, or when they have no easy means of communication (e.g., interactions between algorithms and humans^25^).

Some of these limitations can be overcome by *indirect* interaction^26^. In this case, individuals are not directly in contact with one another, but work separately on a common shared group solution. This type of interaction (also known as stigmergy in biological systems) has been heavily investigated in social insects^14,27^. For example, when ants engage in the construction of a nest, individuals adapt their behaviour to the current state of the collective construction, which reflects the cumulative actions of all other ants^2^. Information is therefore exchanged indirectly, via the collective solution, with no need for direct communication between individuals. This principle can also be applied to human groups; a Wikipedia article, for example, emerges mostly as the result of indirect interactions between multiple contributors^9,14^. In the simplest case, indirect interaction takes the form of a transmission chain, where group members work on a problem sequentially, one after another^28^. Each individual starts from the final solution of her predecessor and tries to improve it, hence gradually giving rise to a collective solution that accumulates the contributions of all group members. Transmission chains have traditionally been investigated in the context of cultural evolution^28–31^ and have more recently been applied to other domains^26,32,33^.

Beside all these methods, there exists a second class of approaches that do not involve any form of information transfer between individuals. In these cases, individuals first solve the same problem independently and in isolation, then their solutions are eventually combined by an external entity to produce the collective outcome^10^. The most prominent example of such procedures is the *wisdom of crowds* in which individual solutions are merged by means of a statistical aggregation function, such as the mean or the median of all solutions^7^. Wisdom of crowds methods are easily scalable as they allow for arbitrarily large group sizes and can yield to accurate solutions^7,34,35^. One drawback, however, is that most statistical aggregation techniques cannot be easily applied to multi-dimensional solutions, such as when optimizing a protein folding configuration^36,37^, improving quantum transport techniques^38^, or trying to solve a jigsaw puzzle^39^. Thus for problems that have a multi-dimensional solution structure, the most common practice consists in collecting a large number of independent and hence diverse solutions and choose the best one at the end^36,38^.

In this work, we specifically focus on problems that have such a multidimensional solution structure. How should a group of given individuals be structured in this case? In particular, we compare two types of methods: Groups that work on the collective solution sequentially, such as transmission chains, or groups that work on independent solutions in parallel? Consider, for instance, the traveling salesman problem – an optimisation task where one has to find the shortest path connecting all cities on a map exactly once^40^. In a transmission chain, the first individual proposes her solution and transfers it to the next person, who will try to optimize it and pass it in turn to the third one, and so forth. This process continues until all individuals of the group have worked on the collective solution. Would the emerging collective solution of the group be better or worse than when letting all group members search independently and choose the best one at the end?

In the present paper, we compare the performance of these two methods by means of a behavioural model and a dedicated experiment. In particular, we study how the collective performance, of both methods are influenced by (1) the complexity of the problem, (2) the individual’s degrees of freedom used as a proxy for the individual’s skill, (3) the group size, and (4) the group’s diversity.

**Figure 1:**
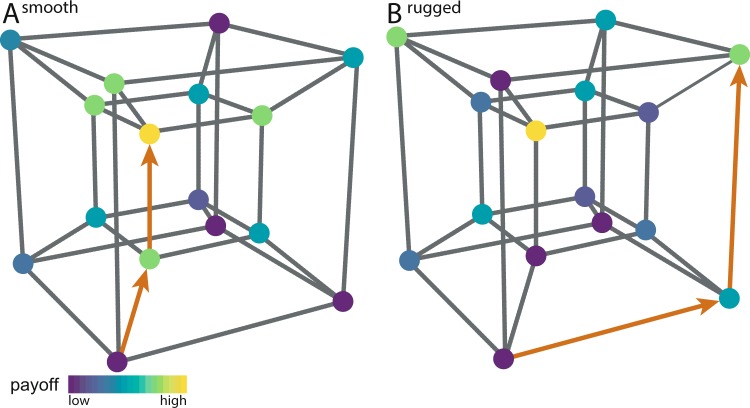
Two examples of four-dimensional NK-landscapes (*N* = 4). Each dot represents one solution and the links between the dots indicate a change along one dimension, illustrating that one can directly move from one solution to another. The color-coding indicates the payoff associated with each solution. In (**A**), the landscape is smooth (*K* = 1) and has one single locally and globally optimal solution (in yellow). The search trajectory (in red) illustrates how a searcher could reach the optimal solution by gradually moving to the highest neighboring solution. In (**B**), the landscape is rugged (*K* = 3). It has numerous local optima (i.e. solutions where all neighboring solutions give a worse payoff) and a simple exploration strategy based on gradual improvements is likely gets stuck on a sub-optimal solution.

To address these questions, we model problem-solving as a search task^41,42^. We assume that individuals are searching for the best possible solution in a multi-dimensional NK-landscape representing the solution space (see Fig. 1 and ref. ^43^). For the transmission chains, individuals search sequentially, one individual after another, each one starting from the last position of her predecessor. The collective solution is then given by the last person’s final position in the landscape. For independent solvers, all individuals start at the same initial position and search in parallel without interactions. The collective solution is then given by the best final solution of all individuals.

Furthermore, we manipulate two variables: the individual’s degrees of freedom *DoF* and the ruggedness of the landscape *K*. We define *DoF* as the number of dimensions a given individual is able to manipulate (with *DoF* ≤ *N*, *N* being the total number of dimensions of the NK-landscape). *DoF* can, to some extent, constitute an aspect of the individual’s skill. That is, an individual with a higher *DoF* is more flexible in her decisions and has the potential to achieve a higher performance than an individual with low *DoF*. For example, an individual with *DoF* = 2 searching in a NK-landscape with *N* = 10 can only manipulate two out of the ten dimensions and can hence only search a restricted area of the landscape, whereas an individual with *DoF* = 10 can potentially search the entire landscape.

The parameter *K* represents the ruggedness of the landscape, i.e. the number of local optima, and is used as a measure of the problem complexity (see the methods section for more details). Smooth landscapes (with low values of *K*) are reminiscent of real-world problems that are well understood and as such can be easily solved with gradual optimization. In contrast, rugged landscapes (with high values of *K*) have a noisier structure. Such landscapes can be interpreted as unstructured or less understood real-world problems where gradual optimization is usually not an efficient strategy (i.e., it easily gets stuck on local optimal solutions^44,45^).

## Results

### Numerical simulation

**Figure 2:**
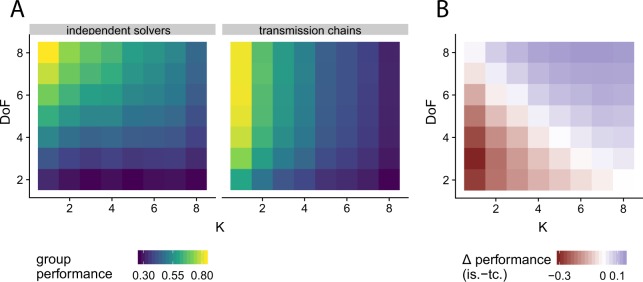
Performance for transmission chains and independent solvers as obtained by numerical simulation, for a group size of 8 individuals and *N* = 10 dimensions. (**A**) Average collective performance of the two methods for varying degrees of complexity *K* and individual’s degrees of freedom *DoF*. (**B**) Difference in performance between the two methods. Positive values, in blue, indicate that independent solvers outperform the transmission chains and vice versa, in red.

We first propose a heuristic model to describe how individuals search in multidimensional landscapes. For this, we assume that individuals randomly, without memory of their past decisions, manipulate one dimension of their current solution and switch to that new solution if it produces a better payoff than the current one^46,47^. We use this model to simulate transmission chains and independent solvers, while systematically varying the complexity of the problem *K*, and the individual’s degrees of freedom *DoF*. As shown in Fig. 2A, both methods are influenced by *K* and *DoF*. As intuitively expected, performance decreases with increasing problem complexity, and increase with increasing *DoF*. However, the transmission chains are less sensitive to the *DoF* than the independent solvers, giving rise to two zones of interest as shown in Fig. 2B: (1) In the lower left corner – for rather smooth problems and individuals with a low degree of freedom – transmission chains outperform independent solvers, (2) in the upper right corner – for rather rugged problems and individuals with a high degree of freedom – groups of independent solvers perform better. Between these two zones, the performance of both methods become increasingly similar.

### Experimental data

**Figure 3:**
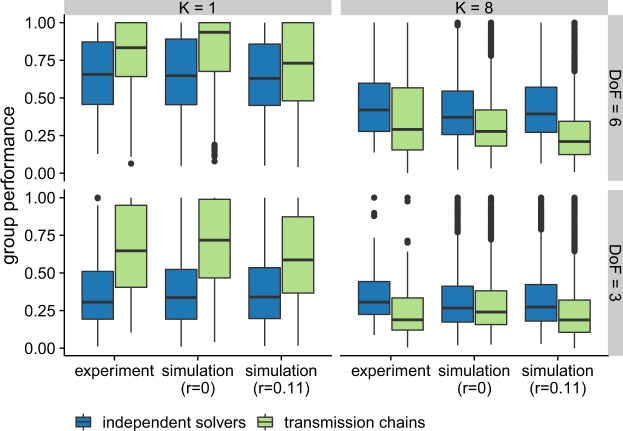
Observed and simulated performance for the transmission chains (in green) and the independent solvers (in blue), for smooth and rugged landscapes (as columns, *K* = 1 and *K* = 8) and for low and high individual’s degrees of freedom (as rows, *DoF* = 3 and *DoF* = 6). The box plots indicate the interquartile range (box), the median (horizontal line) and 1.5-times interquartile range (whiskers). Outliers are shown as a single dot. For the simulations, the parameter *r* indicates the probability of risky decisions (i.e. the probability to leave a solution for a worse one). The group size is limited to eight individuals.

Our simulations suggest that the complexity of the problem and the individual’s degrees of freedom determine which of the two methods performs better. We verify this prediction by means of a controlled experiment. In the experiment, participants searched for the best possible solution either in smooth or rugged landscapes (*K* = 1 or *K* = 8, respectively), and with low or high degrees of freedom (*DoF* = 3 or *DoF* = 6, respectively). Participants were either part of a transmission chain or in a group of independent solvers (see the Methods section for the detailed procedure). As shown in Fig. 3, our experimental data confirm the model predictions. In smooth environments, transmission chains outperform independent solvers (*t*(338) = −6.56, *p* ≤ 0.005, 95ci:  −258.17–139.09, BF  > 100), and this difference is larger for low *DoF* (*t*(166) = −7.07, *p* ≤ 0.005, 95ci:  −361.92–203.90, BF  > 100) than for high *DoF* (*t*(172) = −2.89, *p* ≤ 0.005, 95ci:  −191.24 – −36.11, BF= 15.03). In rugged environments, the opposite is true: Independent solvers outperform transmission chains (*t*(336) = 4.02, *p* ≤ 0.005, 95ci: 51.00 − 148.43, BF  > 100). However, contrary to the predictions, the difference of performance is not larger for higher *DoF* (*t*(168) = 2.86, *p* ≤ 0.005, 95ci: 32.44–176.53, BF = 13.98) than for for lower *DoF* (*t*(168) = 2.93, *p* ≤ 0.005, 95ci: 30.56–156.87, BF = 16.48). In other words, our simulations predicted that the transmission chains would perform better for rugged (i.e., more complex) problems than they actually do. Why is that so?

In transmission chains, performance is considerably affected by the decisions of the last individuals^26^. For instance, the last person of a chain could make the decision to leave a good solution found by her predecessors and search for a better one. A failure to find such a better solution would impair the collective performance of the entire group (which is determined by the solution of the last individual only).

**Figure 4:**
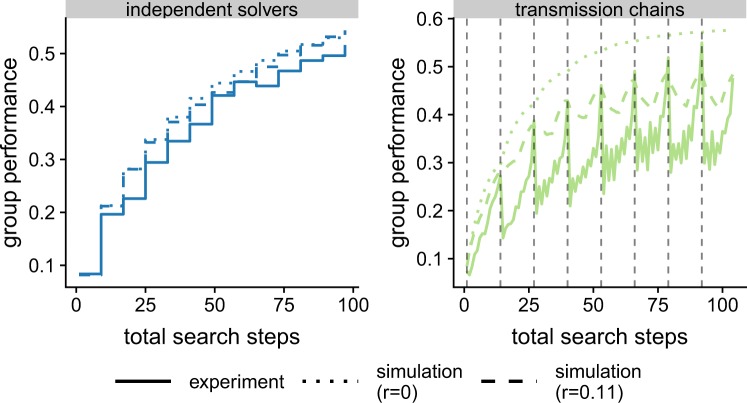
Group performance as a function of total search steps for *S* = 6 and *K* = 1 or 8. Total search steps are the sum of all search steps at the disposal of the entire group. For the transmission chain, a dashed vertical lines indicates that a new individual has started.

As our simulated agents only leave a solution for a better one, our model fails to predict decisions that lead to payoff decrease (see Fig. 4). To capture such decisions and enable a better fit to the experimental data, we extended our model with a parameter *r*: the ratio of risky decisions. The parameter *r* reflects the probability that an agent does not immediately return to its previous solution after sampling a worse one. That is, for *r* = 0 the behavior of the agents is identical to the search model without the parameter, whereas for *r* = 1 the agents behavior is not guided by the payoff at all. Hence, *r* allows some flexibility in the behavior of the agents, preventing them from getting stuck at only locally optimal solutions, but at the same time increases the risk of losing track of a previously found solution (see Supplementary material, Fig. 3 for the influence of *r* on the performance of the different methods).

We fit *r* to our experimental data. On that account, we only consider decisions that lead to a worse solution (i.e. a decrease in payoff) and measure the ratio of cases where participants have not immediately returned to their previous solution (i.e. have not reverted their decision). The fitted value of *r* = 0.11, indicates that in 89% of all cases participants immediately return to their previous solution when a worse payoff is encountered. The new simulations match the observed performance very closely (see Figs. 3, and 4). That is, *r* > 0 decreases the performance of transmission chains for high complexity and low *DoF*, while slightly improving the performance of groups of independent solvers.

### Group size and diversity

**Figure 5:**
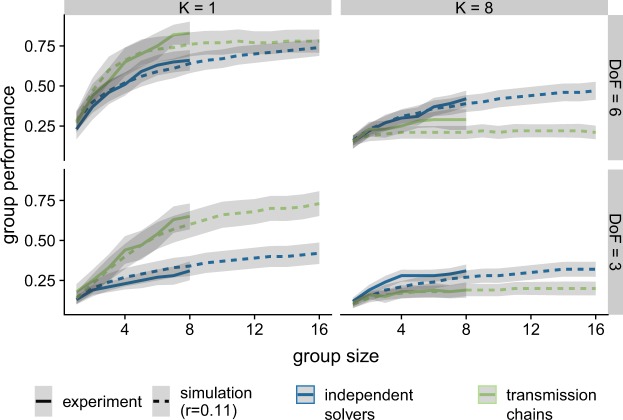
Influence of group size on performance for the two procedures (color-coded), as observed in the experiment data and obtained in simulations. Error-bands indicate the variance of group performance.

We investigate the influence of group size for the two methods. To account for smaller groups in the experimental data, we simply excluded later individuals to match the desired size and recalculated the group’s performance. Overall, simulations and experimental data exhibit very similar tendencies (see Fig. 5). In either case, our previous findings are robust to group size variations: with at least three group members, transmission chains outperform independent solvers in smooth environments and are outperformed in rugged ones. In general, group performance increases with group size while the difference between the two procedures remains about the same. The only exception concerns smooth landscapes with a high *DoF*, where both strategies’ performance tend to become more similar.

In line with extant research^48^, we find a diminishing returns for larger groups. In other words, performance does not improve linearly with group size, but eventually plateaus. For example, over all conditions, an increase in group size from two to five members improves performance by an average of 0.1 in the experimental data, whereas the same change from five to eight members only improves performance by 0.07. In transmission chains, too many members can even decrease the collective performance, because longer chains increase the risk of losing a good solution due to a risky decision (as described by the parameter *r*).

Finally, we study how the collective performance is impacted by the group diversity – a factor that is known to be critical for collective intelligence^35,49–51^. For this, we define a group’s diversity as the dissimilarity between the dimensions that the group members can manipulate. Put differently, a diverse group is made of individuals that have different perspectives on the same problem. Formally, diversity is measured as the average number of dimensions that only one individual can manipulate in all possible pairs of individuals in the group.

**Figure 6:**
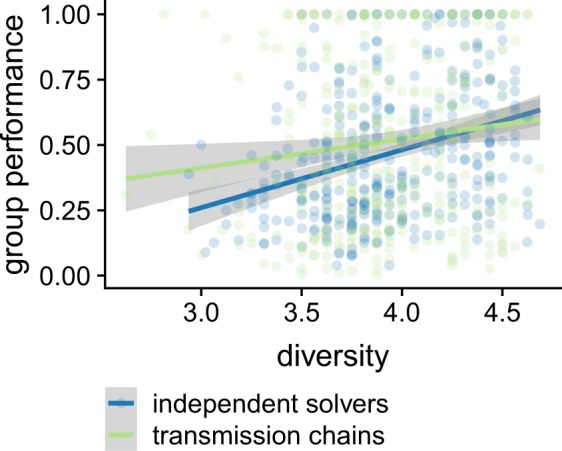
Influence of diversity on the collective performance, as observed in our experimental data. Diversity is defined as the dissimilarity between the dimensions that the group members can manipulate. Each point corresponds to one landscape in one condition. The straight lines indicate the best fitted linear models and the standard error (color-coded).

As shown in Fig. 6, our results reveal very strong evidence for a positive influence of diversity on the performance of independent solvers (*F*(1, 346) = 39.15, BF  > 100, *adj*. *r*^2^ = 0. 10) and a moderate one for transmission chains (*F*(1, 343) = 5.47, *BF* = 3.14, *adj*. *r*^2^ = 0. 01). In short, diversity is indeed a positive factor, and more diverse groups outperform less diverse ones, irrespective of the chosen method.

## Discussion

How should a group of individuals be structured to find the best possible solution to multidimensional problems? Here we compared two approaches: (1) transmission chains, where individuals tackle the problem sequentially, one after the other, each one building on the solution of its predecessor, and (2) groups of independent solvers, where individuals tackle the problem in parallel without influence and the best solution found in the group is selected afterwards. Our results suggest that the performance of the two methods depend on the interplay between two factors: the problem complexity (i.e., the ruggedness of the landscape) and the individual’s degrees of freedom *DoF* (i.e. the number of dimensions an individual can manipulate). Transmission chains outperform groups of independent solvers for easy, smooth problems or when individuals have low degrees of freedom. However, independent solvers have a better performance for complex, rugged problems or when the group members have high degrees of freedom. To put it differently, when trying to continuously improve a solution to a well understood problem or when dealing with inflexible and hence potentially unskilled individuals, reliance on previous solutions is beneficial. When trying to come up with solutions to an unstructured or ill defined problem in a group of experts one should rather select amongst multiple independent suggestions.

The intuition underlying these results is the following. In smooth landscapes (i.e., easier problems), the global maximum can be found by means of a simple hill-climbing strategy that operates on all the problem’s dimensions. However, individuals with low degrees of freedom have only access to a subset of these dimensions. For that reason, independent solvers performs poorly in this case (corresponding to the lower-left corner of the map, Fig. 2B). Transmission chains, in contrary, combine the dimensions that group members can manipulate. The different dimensions of the problem can therefore be optimized sequentially, explaining the better performance of this method here. As the group members have a higher *DoF* (i.e., moving towards the upper-left corner of the map, Fig. 2B), the difference between the two methods decreases. The collective outcome becomes naturally less sensitive to the chosen method when smooth problems are addressed by highly flexible individuals.

As the problem becomes more complex, the performance of both methods decline, but the decrease is less pronounced for groups of independent solvers. The challenge of such rugged environments is that the hill-climbing strategy, which most participants follow, gets easily stuck at a local optimum. In this situation, independent searchers exhibit better performance because individuals in such groups try different trajectories and arrive at different solutions – thus maximizing the likelihood that at least one of them reaches a good solution. Along these lines, citizen science projects, where people try to solve extremely complex optimization problems, have shown that high-performing participants are less efficient when first exposed to example solutions than when they work independently^36,38^.

Our results are mainly driven by the fact that most individuals rely on a hill-climbing strategy. This behavior, which avoids any decrease in payoff, can be interpreted as risk aversion^52^. Research has shown that the willingness to take risks is decreased when the problem space becomes larger and more complex^53,54^. While the decrease in risky decisions prevents individuals from "getting lost” in very large problem spaces, it also hinders the discovery of *leaps* – truly novel and substantially improved solutions^55^. To avoid this effect one might investigate the influence of adding a "safety net” – for instance by rewarding the collective performance – which could possibly increase the frequency of riskier search behaviors. In situation, where the individuals search is not predominantly guided by payoff (e.g., when individuals are more likely to take risky decisions and move away from a local optimum, or when the payoff information is not immediately available), additional simulations indicate that transmission chains would outperform independent searchers (see Supplementary material, Fig. 2).

In our simple implementation of transmission chains, the collective performance depends substantially on the last individuals of the chain. In other words, the system has no memory of the past solutions, which can result in losing track of a very good solution^26^. This leads to very volatile collective performance over time, as observed in our experimental data and when introducing risky decisions in the simulations (see Fig. 4). This is along the lines of previous research showing that less inclusive strategies, i.e. strategies that depend on a smaller number of individuals, are more prone to wrong judgments, outliers and noise^7,34^. Nevertheless, research in cumulative cultural evolution – studying how an innovation can emerge as solutions are passed from person to person, across generations – has shown that more sophisticated forms of transmission chains can retain memory of past events and yield more stable collective results.

Our findings strengthen the connection between collective problem-solving and cumulative cultural evolution^56^. In addition to our main results, we find behavioural patterns similar to those that have been observed in cultural evolution research, such as the diminishing returns for larger group sizes^48^, the influence of diversity on group performance^49^ and the impact of collective problem-solving methods on group success^44,57^. Our results complement these findings by comparing the influence of different methods when controlling for the total number of search steps (as proposed by^56^) and when systematically manipulating the individual’s degrees of freedom as a proxy for the individual’s skill.

Future research will consider mixtures and variations of collective problem-solving methods, such as alternating phases of influenced and independent search^58^, comparing direct and indirect interactions, or mixing direct and indirect interactions in more elaborated chain structures^59^.

## Methods

### Search environment

The search environments used in our simulations and the experiment were produced by means of the NK-model, which generates multi-dimensional tunably rugged landscapes^42,47^. The structure of these landscapes is determined by the two eponymous parameters: *N* is the number of binary dimensions and *K* controls the ruggedness by varying the number of interdependencies between each dimension. Low values of *K* generate smooth landscapes with few or no local maxima, which are easy to solve by means of a local optimization procedure (i.e. hill climbing). In contrast, high values of *K* create rugged landscapes with many local maxima, where local optimization is not an efficient search strategy (see Fig. 1 for a visualization of two NK-landscapes and^43^ for a more detailed description of the underlying model).

The NK-landscapes used in our study were generated by fixing *N* = 10 (i.e. our landscapes have 10 binary dimensions corresponding to 2^10^ = 1024 different solutions) and with various values of *K* (see Supplementary material, Fig. 4 for the influence of different values of *N*). Following several authors, we normalized the payoffs in each landscape by dividing them by the maximal achievable payoff and using a monotonic transformation to raise each payoff to the power of eight^42,47^. This process causes most solutions to be mediocre and only few to be very good.

### Simulation procedure

Following^47^, we use a minimalistic heuristic model of individual search (which nevertheless captures experimental data surprisingly well). The model assumes that each agent manipulates one randomly selected dimension at a time, and moves to the new solution if it offers a better payoff than the current one. The agent repeats this search behaviour until the end of the search time. We vary the individual’s degrees of freedom by allowing only a limited number of randomly selected dimensions to be manipulated by the agent. For example, for *DoF* = 2 the agent can only manipulate two dimensions of the search environment.

The duration of the search is set to 16 consecutive decisions, but our results seem robust to variations of this number (see Supplementary material, Fig. 5 for the influence of search duration on performance). All results are averaged over 2.500 repetitions.

### Experimental treatment

Participants were instructed to search for the best possible solution in a NK-landscape. To facilitate the visual representation, all payoffs were multiplied by 1, 000, and the 10 dimensions of the landscape were represented as 10 light bulbs that could be either on or off (representing the binary values ‘0’ or ‘1’, see Supplementary material, Fig. 1). Not all light bulbs could be manipulated, due to the restrictions imposed by *DoF* (those were visually marked by a cross). In each round participant could change the state of one light bulb. After their decision, they were informed about their new payoff and were allowed to return to their previous solution before a new round started.

The eight experimental conditions were selected based on preliminary simulation results, and were matched to the four corners of the Fig. 2B. The eight conditions consisted of smooth and rugged landscapes (*K* = 1 or *K* = 8, respectively), low or high individual’s degrees of freedom (*DoF* = 3 or *DoF* = 6, respectively) and transmission chain or independent group. The order of the experimental conditions was randomized. Each participant played a total of 128 levels, that is, 16 landscapes per experimental condition. To prevent participants from searching all possible solutions, the duration of the search was limited to 2 × *DoF* rounds for all experimental conditions. Groups of eight individuals were formed searching the same landscape in the same condition. In the transmission chains, an individual player can never receive its own final solution as a new starting solution.

### Experimental procedure and participants

Participants were recruited from the Max Planck Institute for Human Development’s pool and gave informed consent to the experiment. The experimental procedure was approved by the Ethics Committee of the Max Planck Institute for Human Development and was performed according to the Declaration of Helsinki. Participants were first familiarized with the experiment and informed about their goal, the incentives, and the rules of search in the experiment. Supplementary material, Fig. 1 shows the experimental interface.

We invited 50 participants to the behavioural laboratory of the Max Planck Institute for Human Development. Data of two participants had to be excluded due to technical issues. There were 25 females among the remaining 48 participants (mean age  = 27.9, *SD* = 5.13). Participants received a flat fee of 8 € plus a monetary bonus based on their total performance (0.16 € per 1000 points, mean bonus = 6.65 €, *SD* = 1.11€). The average completion time was 33.64 minutes (*SD* = 10.67 minutes).

### Statistical tests

All reported t-tests are one sided, as we tested directional hypothesises derived from the simulations results. We also report Bayes factors (BF), quantifying the likelihood of the data under *H*_1_ relative to the likelihood of the data under *H*_0_. For parametric tests, the data distribution was assumed to be normal, but this was not formally tested^60^. Our hypotheses also hold for non-parametric Wilcoxon ranked sum tests.

## Supplementary information

Supplementary materials.

## Data Availability

Anonymous participant data, model simulation, and experimental code are available at https://github.com/cuehs/transmissionchains_independentsolvers
