# Supplementary material for: Comparing Groups of Independent Solvers and Transmission Chains as Methods for Collective Problem-Solving
Source: Sci Rep. 2020 Feb 20;10:3060. doi: 10.1038/s41598-020-59946-9 (PMC7033214; doi:10.1038/s41598-020-59946-9)
Supplement: Supplementary file 1 — Supplementary materials. [file 41598_2020_59946_MOESM1_ESM.pdf]

Supplementary materials for *Comparing Groups of Independent Solvers and Transmission Chains as Methods for Collective Problem-Solving*

Kyanoush Seyed Yahosseini and Mehdi Moussaïd
Center for Adaptive Rationality, Max Planck Institute for Human Development
Berlin, Germany

2020

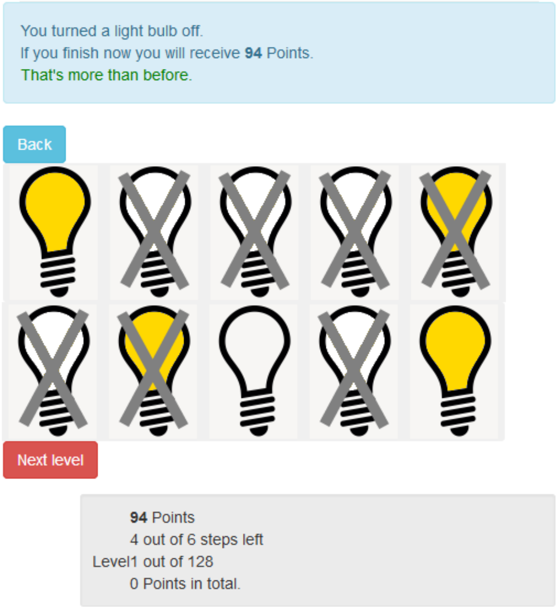

Figure 1: Experimental interface (translated from German to English). The ten light bulbs correspond to the ten dimensions of the the NK-landscape, and the state of each light bubble ('on' or 'off') represents the values 1 and 0. Grey crosses indicate dimensions that cannot be manipulated due to the restrictions imposed by DoF ($DoF = 3$ in this example). Information about the experiment, such as total number of points and remaining number of landscapes ('level') are provided in the grey box at the bottom. The blue box at the top shows information related to the previous decision. In each round, participants could either change the state of one light bulb (by clicking on the desired one), skip the remainder of a level if satisfied (by clicking the 'next level' button), or return to their previous solution (by clicking the 'back' button).

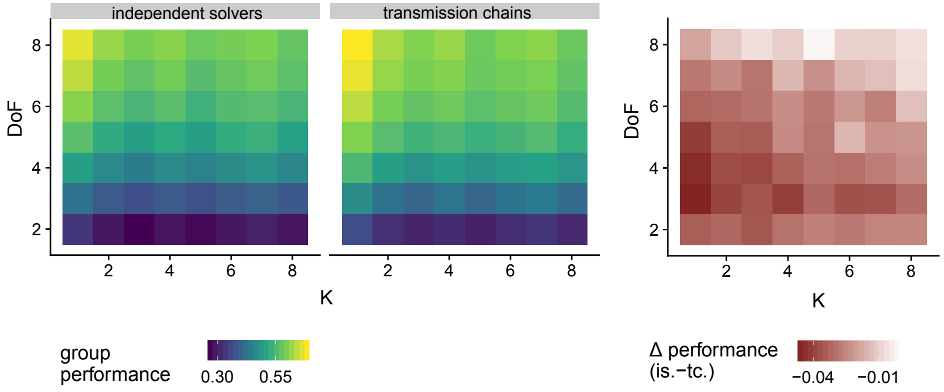

Figure 2: Performance of the transmission chains and groups of independent solvers with a rate of risky decisions $r = 0.8$ and reporting the best solution found. (A) Group performance for varying degrees of complexity K and individual's degrees of freedom DoF. (B) Difference in performance between the two methods. Positive values indicate that independent groups outperforms the transmission chain, and vice versa.

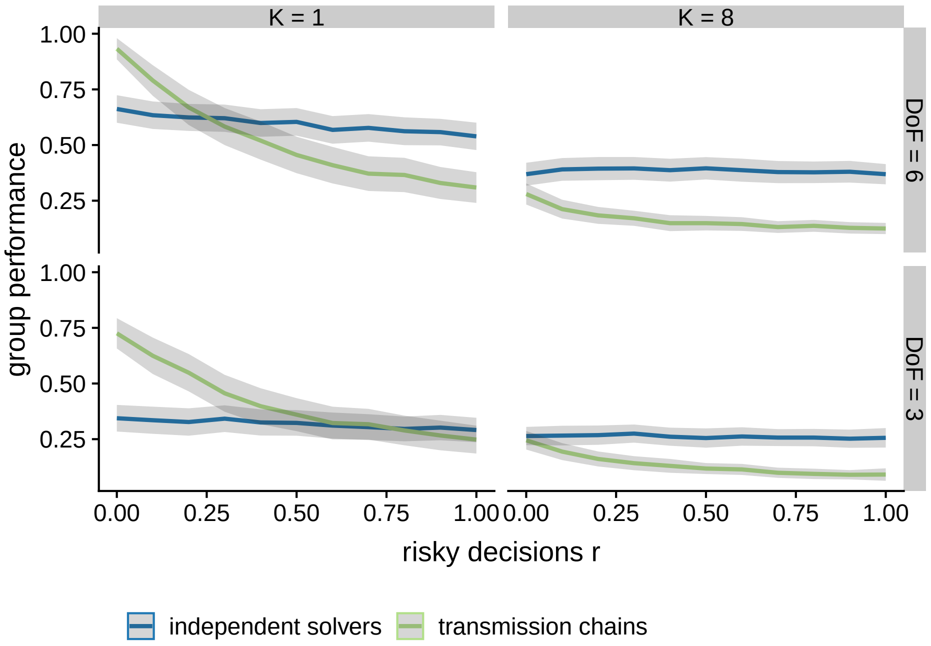

Figure 3: Influence of the ratio of risky decisions r on performance for the two procedures (color-coded), as obtained in simulations.

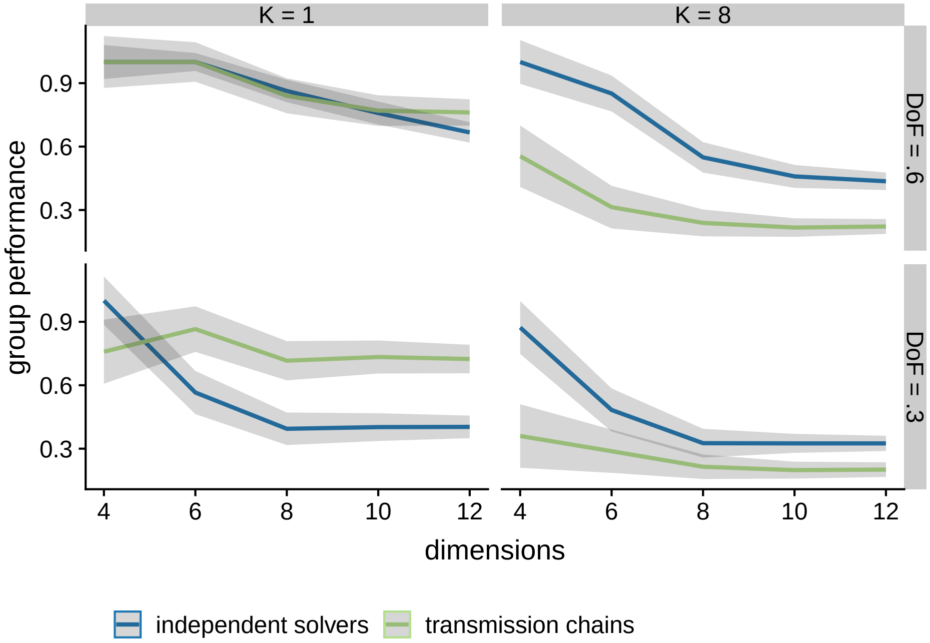

Figure 4: Influence of the dimensions of the problem-space N on performance for the two procedures (color-coded), as obtained in simulations. The number of possible solutions is 2^N. Here we define individual's degrees of freedom DoF as the the rounded fraction of the N dimensions an individual can manipulate (e.g. DoF of .6 corresponds to 7 dimensions in the $N = 12$ case and to 2 dimensions in the $N = 4$ case.)

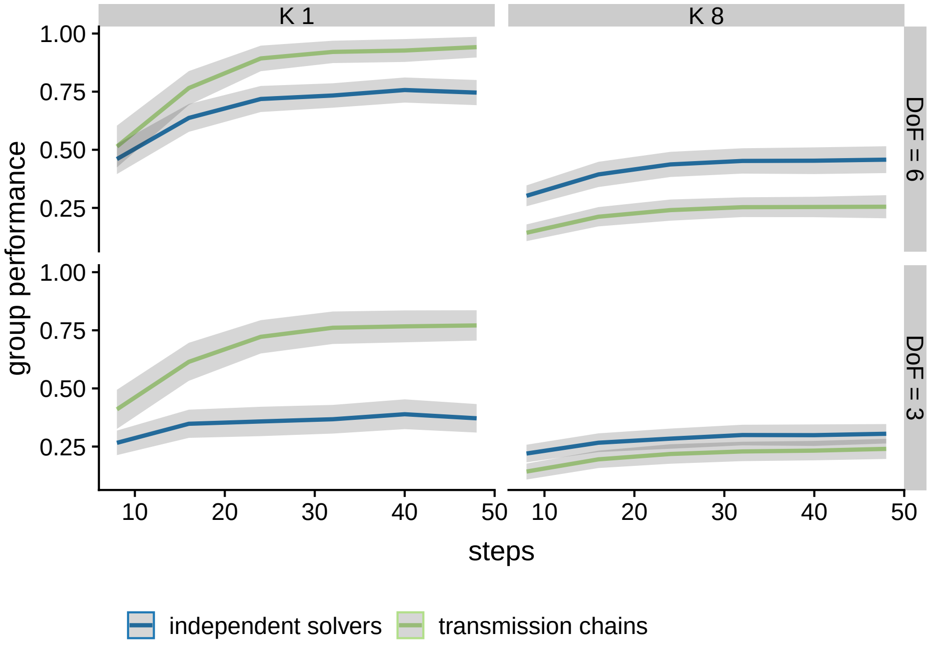

Figure 5: Influence of the search duration (steps) per individual on performance for the two procedures (color-coded), as obtained in simulations.
